# New Journal

**Published:** 1856-01

**Authors:** 


### BIBLIOGRAPHICAL NOTICES.

*New Journal.*—We have received the first number of the "Medical Observer," Cincinnati.

This journal is welcomed into our midst with hearty good will, and promises to become an interesting and companionable member of our present large family. The number now before us, shows that we have a good reason for the hopes that we entertain for its future efficiency. We cheerfully welcome it to our side of the circle, that is the Western side, and can assure it of our warm regard so long as it continues to bear its part so well. It must expect trials and difficulties, disappointments and mortifications, but it will learn that perseverance, labor, and energy will overcome all when "we know we are right." Our advice has certainly not been required, but voluntarily and most respectfully we would suggest the use of a free dose of *tart. ant.*, and the free inunction of *ung. hydrarg.*, to those *malignant* institutions, which, covered with foul leprosy, are disseminating disease and death broadcast through the land.

